# Olfactory epithelium changes in germfree mice

**DOI:** 10.1038/srep24687

**Published:** 2016-04-19

**Authors:** Adrien François, Denise Grebert, Moez Rhimi, Mahendra Mariadassou, Laurent Naudon, Sylvie Rabot, Nicolas Meunier

**Affiliations:** 1 NBO, UVSQ, INRA, Université Paris-Saclay, F-78350 Jouy-en-Josas, France; 2 NBO, INRA, Université Paris-Saclay, F-78350 Jouy-en-Josas, France; 3 Micalis Institute, INRA, AgroParisTech, Université Paris-Saclay, F-78350 Jouy-en-Josas, France; 4 INRA, UR1404 MaIAGE, F-78350 Jouy-en-Josas, France; 5 Micalis Institute, INRA, AgroParisTech, CNRS, Université Paris-Saclay, F-78350 Jouy-en-Josas, France

## Abstract

Intestinal epithelium development is dramatically impaired in germfree rodents, but the consequences of the absence of microbiota have been overlooked in other epithelia. In the present study, we present the first description of the bacterial communities associated with the olfactory epithelium and explored differences in olfactory epithelium characteristics between germfree and conventional, specific pathogen-free, mice. While the anatomy of the olfactory epithelium was not significantly different, we observed a thinner olfactory cilia layer along with a decreased cellular turn-over in germfree mice. Using electro-olfactogram, we recorded the responses of olfactory sensitive neuronal populations to various odorant stimulations. We observed a global increase in the amplitude of responses to odorants in germfree mice as well as altered responses kinetics. These changes were associated with a decreased transcription of most olfactory transduction actors and of olfactory xenobiotic metabolising enzymes. Overall, we present here the first evidence that the microbiota modulates the physiology of olfactory epithelium. As olfaction is a major sensory modality for most animal species, the microbiota may have an important impact on animal physiology and behaviour through olfaction alteration.

The influence of microbiota on animal physiology is now well established. This influence ranges from providing essential nutriments such as vitamin K^1^ to the modulation of social behaviour^2^. The study of germfree animals has been critical to decipher the importance of the microbiota and one of the most spectacular differences between conventional and germfree animals concerns the intestinal epithelium physiology, which is greatly altered in germfree animals^3^. Combined with the finding that the gut harbours the most abundant host microbial ecosystem^4^, these are the reasons why studies on interactions between epithelia and microorganisms have mainly focused on intestinal epithelium.

Among the different epithelia of vertebrate organisms, the olfactory epithelium (OE) has been completely overlooked with regards to a potential role of microorganisms on its development and on efficiency of odorant transduction. Yet, a recent study examined the presence of microbiota in different areas of the nasal cavity, one being located next to the OE. An important diversity of microorganisms cohabits in this area with variations between zones of the nasal cavity^5^. Another study points out that microbiota composition of the nasal cavity differs globally much more between individuals than other microbiota^6^.

The olfactory system plays a major role in the feeding behaviour of humans^7^, but also in social behaviour in most animal species^8^. The first step of odorant processing takes place in the OE, which is divided into several subsystems. In mammals, the main OE is localized in the nasal cavity, positioned around a modified part of the cranial bone, the cribriform plate, which allows the passage of the olfactory nerves to the central nervous system. It holds olfactory sensory neurons (OSN), which possess cilia extruding into a mucus layer in contact with the environment. The mucus contains olfactory binding proteins that may act as carriers of hydrophobic odorants into the hydrophilic mucus, allowing them to reach the OSN cilia^9^.

Each OSN expresses only one olfactory receptor randomly selected among a large multigene family of G-protein coupled receptors^10^. When activated by odorants, most OR activate in turn the adenylate cyclase III through the G protein G_olf_. The subsequent rise in cAMP level depolarizes the OSN through activation of a cyclic nucleotide gated channel. Odorants are eliminated from the mucus through xenobiotic metabolising enzymes, present both in the mucus and in the olfactory epithelium cells^9^.

OSNs are renewed throughout the life of the animal^11^. Indeed, as OSNs are in direct contact with the environment, they are permanently exposed to aggression from oxidative stress, pathogens or xenobiotics and thus have a limited life expectancy.

Microbiota could affect most of the first steps of odorant detection. This potential influence could arise from the microbiota present in the OE mucus, impacting the OE structure by close interactions with it, as observed with the gut microbiota and the intestinal epithelium. The gut microbiota could also act on the OE remotely, through the release of metabolites subsequently absorbed by the intestinal epithelium and circulating in the body^12^. Microbiota influence on the OE structure may involve a modulation of the OSN turn-over, which, in turn, may change the OSN population properties. As young neurons possess different odorant transduction properties^13^, it could lead to alterations in odorant detection. Microorganisms present in the OE mucus could also change the accessibility of odorants to the OSN cilia, as well as the rate of odorant degradation by metabolising them or influencing the xenobiotic metabolising enzymes expression in the OE. Finally, OE or gut microbiota could influence olfactory signaling by releasing odorants, as it has been shown that microbiota participates in the production of odorants related to social interactions^14^.

To examine the influence of microbiota on olfaction, we compared germfree and conventional, specific pathogen-free, mice. First, we ascertained the presence and analysed the abundance and diversity of the microbiota on the OE surface, using 16S rDNA sequencing. Then, we examined the OE anatomy and cellular dynamics and measured the OE responses to odorants using electro-olfactogram recordings (EOG). EOG allows to record the receptor potential of an OSN population induced by odorant stimulation^15^, reflecting all events related to the latency of odorants to reach OSN cilia, the transduction pathway, and the OE ability to clear odorants. Finally, to explain the EOG differences between germfree and conventional animals, we performed qPCR analysis of genes involved in olfaction transduction pathway and odorant clearance.

## Results

### Characteristics of olfactory epithelium microbiota

**Figure 1:**
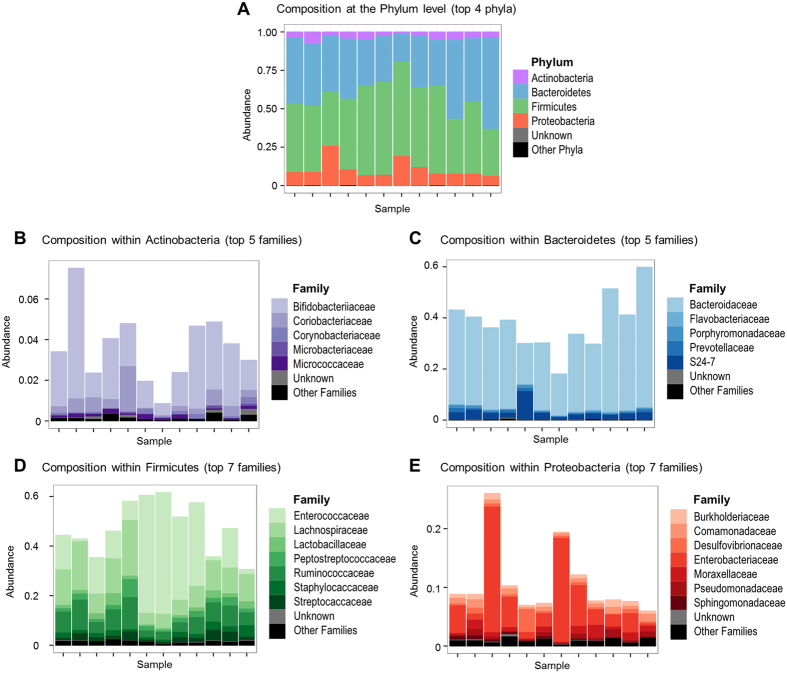
Taxonomic diversity of the microbiota associated with the mouse olfactory epithelium. Analysis was based on 16S rDNA sequencing. Bar graphs show the relative distribution of phyla (**A**) and of families within the most abundant phyla (**B–E**) in the olfactory epithelium samples collected from conventional, specific-pathogen free, mice (n = 12).

Analysis of the 16S rRNA gene revealed the existence of a microbial community associated with the olfactory epithelium and gave an indication of its taxonomic structure. The olfactory epithelium microbial community of the conventional, specific pathogen free, mice was mainly dominated by 4 phyla: Bacteroidetes and Firmicutes accounted for 15% to 60% and 30% to 70% of the overall community, respectively; Proteobacteria and Actinobacteria accounted for 5% to 25% and less than 10%, respectively; other phyla were below 1% (Fig. 1A). The variability across individuals at the phylum level was also found at the family level, although a few number of families were dominant within each phylum, regardless of the individual (Fig. 1B–E). *Bacteroidaceae* markedly dominated the *Bacteroidetes* phylum; *Enterococcaceae*, *Lachnospiraceae* and *Ruminococcaceae* were the major Firmicutes families; and *Enterobacteriaceae* and *Bifidobacteriaceae* dominated the Proteobacteria and Actinobacteria phyla, respectively.

### Anatomy of olfactory epithelium is globally preserved in germfree animals

**Figure 2:**
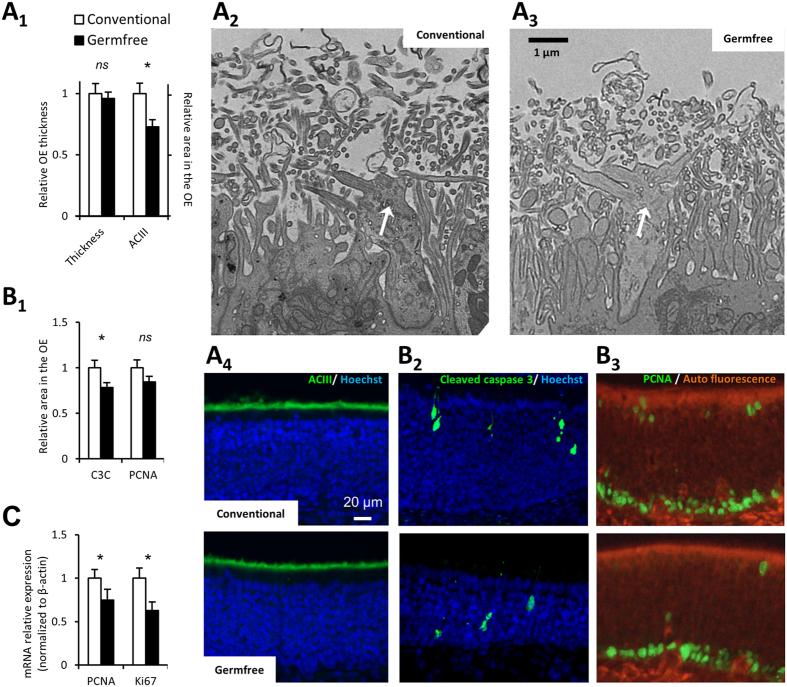
Thinner cilia layer and reduced turn-over in the OE of germfree animals. (**A**_**1**_) The OE thickness was compared between conventional and germfree animals. The cilia layer thickness was evaluated based on adenylate cyclase III (ACIII) staining, whose expression is restricted to olfactory cilia. Results were expressed as mean of the OE thickness and mean of the ACIII signal area in the OE normalized to conventional animals ± SEM (n = 8). (**A**_**2,3**_) Representative transmission electron images of olfactory dendritic knob (white arrow) and associated cilia layer (x2000). (**A**_**4**_) Representative image of ACIII staining in the OE. (**B**_**1**_) The cellular turn-over of the OE was evaluated by quantifying the areas with cleaved caspase 3 (C3C) and PCNA stainings, taken as indices of apoptosis and proliferation levels, respectively. Results were expressed as mean of the C3C and PCNA signal area in the OE normalized to conventional animals ± SEM (n = 19). Representative image of (**B**_**2**_) C3C staining with OSN ongoing apoptosis and (**B**_**3**_) PCNA staining mainly present in basal cells same after (**B**_**2**_). (**C**) The level of proliferation was further evaluated by quantification of ki67 and PCNA expression on cDNAs from olfactory mucosa. Their expression levels were normalized to that of β-actin and are given as mean ± SEM (n = 7). (*) *P* < 0.05.

As the absence of gut microbiota impairs the development of the intestinal epithelium^3^, we first examined if the global anatomy of the OE was altered in germfree animals. The OE thickness (measured based on nuclear staining) was not different between germfree and conventional mice (Fig. 2A_1_), and transmission electron microscopy (TEM) did not reveal important changes except a thinner olfactory cilia layer in germfree animals (Fig. 2A_2,3_). To confirm this potential reduction in cilia layer thickness, we performed immunohistochemistry against adenylate cyclase III, a major component of the transduction pathway mainly present in the olfactory cilia^16^. We observed a significant decreased ACIII signal in the OE of germfree animals (Fig. 2A_1,4_), consistent with the thinner layer of olfactory cilia observed in TEM.

### Cellular dynamics of the OE is changed in germfree mice

Renewal of the OE throughout life has been linked to its aggressive surrounding environment^11^. Among potential threats, microorganisms could release toxins or trigger inflammation, thus promoting apoptosis^17^. We thus examined whether the cellular turn-over was altered in germfree animals. We first evaluated the OE level of apoptosis, reflected by the presence of cleaved caspase 3, a major component of the apoptosis signaling cascade. We observed a significant decrease of cleaved caspase 3 signal in the OE of germ free animals (Fig. 2B_1,2_). We next examined the OE proliferation rate by immunohistochemistry against PCNA and observed a lower tendency, which did not achieve significance, in germfree animals (Fig. 2B1,3). To examine more closely this tendency, we quantified PCNA and Ki67 mRNA levels by RT-qPCR and found both significantly reduced in germfree animals (Fig. 2C). Overall, these results indicate that the cellular turn-over in the OE is reduced in germfree animals.

### Odorant detection is altered in germfree mice

**Figure 3:**
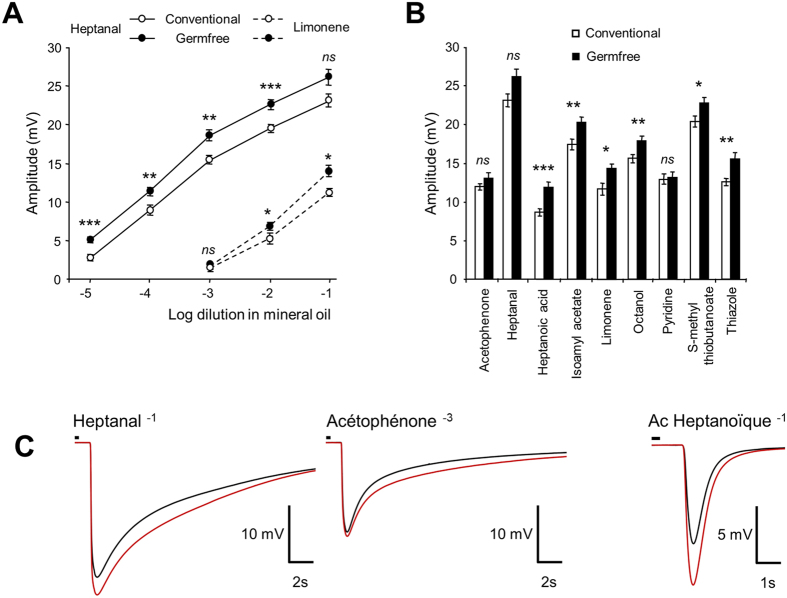
Global increase of responses to odorants recorded by EOG in germfree animals. (**A,B**) EOG responses to various odorants in conventional and germfree animals. Values represent the mean of peak amplitudes ± SEM (n = 12) (*P < 0.05; **P < 0.01; ***P < 0.001). (**C**) Average traces for 3 odorants from conventional (black) and germfree (red) animals. Small black line on top of recordings indicates odorant stimulation.

To assess whether the presence of microbiota could affect the OSN responses to odorants, we performed EOG recordings. We stimulated the OE with increasing concentrations of heptanal and limonene as well as several other odorants. Part of the variation of EOG signal may be based on the microbial metabolism of odorants. We therefore chose odorants with the greatest variety of organic groups and structures (Supplementary Table 1). We measured the maximum amplitude of responses to odorants (Fig. 3) and different kinetics parameters (Supplementary Table 1). The maximum amplitude of responses was significantly increased in germfree animals for all odorants, except acetophenone and pyridine, at all dilutions used (Fig. 3A,B). Despite the higher peak amplitude of the EOG responses, the area of the signal was often not statistically different between conventional and germfree animals, indicating that the response kinetic was affected as well (Supplementary Table 1). Indeed, the depolarization and repolarization phases of the EOG responses were systematically faster in germfree animals, except for the fast component of the repolarization phase in response to acetophonenone 10^−3^. The repolarization phase was less affected than the depolarization without any clear link with odorant structure (Fig. 3C and Supplementary Table 1).

### Expression of genes related to odorant detection in the OE is different between germfree and conventional mice

**Figure 4:**
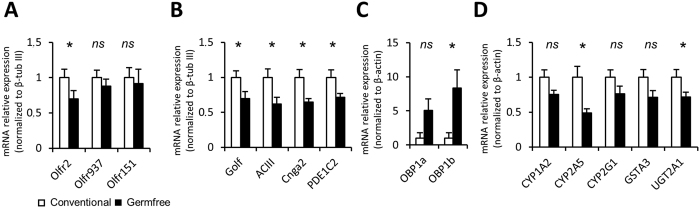
Modulation of the expression of genes related to odorant detection in germfree animals. Quantitative PCR (qPCR) analysis of cDNAs from olfactory mucosa. Genes are grouped by functions; (**A**) olfactory receptors sensitive to odorants tested in the EOG experiment; (**B**) main olfactory transduction pathway components; (**C**) olfactory binding proteins; (**D**) detoxifying enzymes. Their expression levels were normalized to that of β-tubIII (**A,B**) or β-actin (**C,D**) and are given as mean ± SEM (n = 7). (*) *P* < 0.05.

The EOG recordings showed that the presence of microbiota influences odorant detection, we therefore analysed the expression level of various genes related to odorant detection and metabolism, and to olfactory signal transduction. We first examined the level of expression of OR related to the odorants used in the EOG experiments^18^. While the heptanal sensitive Olfr2 expression was significantly decreased, the expression of the heptanoic acid sensitive Olfr937 and of the acetophenone sensitive Olfr151 were not statistically different in the absence of microbiota (Fig. 4A). Most volatile odorants are transduced through the activation of a G protein G_olf_, which in turns activates the adenylate cyclase III (ACIII). The rise in cAMP level in turn activates a CNG channel (Cnga2) and this rise is counterbalanced by the cAMP degrading phosphodiesterase PDE1C2. While we observed a global increase in the EOG signal in response to various odorants in germfree animals, all components of the olfactory transduction cascade were significantly more expressed in conventional animals (Fig. 4B). Odorants are hydrophobic and olfactory binding proteins, among others, are thought to improve the solubilisation of odorants in the nasal mucus, allowing them to reach the olfactory receptors^9^. Because the OE-associated microbiota could interfere with this step, we also examined the level of expression of the two olfactory binding proteins which have been characterised in mice, OBP1a and OBP1b. Both were largely overexpressed in germfree animals, although significance was not reached for OBP1a (p = 0.055), due to an important individual heterogeneity (Fig. 4C). Finally, odorants are rapidly detoxified in the OE through various pathways, involving mainly cytochrome P450 enzymes, gluthathione-S-transferase (GST) and UDP-glucuronosyl transferase (UGT). We examined the expression of the genes encoding the main CYP450, GST and UGT isozymes in the OE^9^. While we observed a global decrease of their expression level in the germfree animals, it reached significance only for CYP2A5 and UGT2A1 (Fig. 4D).

## Discussion

In this project, we wanted to examine the influence of microbiota on olfaction. We observed that, in germfree mice, while the OE thickness was unaltered, the cilia layer was thinner, along with a reduced expression of most genes related to the olfactory transduction pathway. Despite this decrease, we observed a stronger EOG signal amplitude for a wide array of odorants. As the gut bacterial population represents the major part of the microbiota^19^, it may be that the observed change in OE physiology mainly arise from its absence in germfree animals. If so, we would expect a similar evolution of odorant detection changes in germfree animals across odorants. However, EOG recordings are differentially affected according to the odorant nature pointing out that the OE associated microbiota may directly affects the OE physiology.

The microbiota of the upper airways has been relatively unexplored, compared to other body sites such as gut and skin. A few molecular studies have been performed in humans to characterize the microbiota of nares^20^ and nasal cavities^21^. However, to the best of our knowledge, we report here the first description of the bacterial communities associated with the olfactory epithelium, thus contributing to the holistic overview of commensal microbiota. As reported in nares and nasal cavities, the OE microbiota was mainly dominated by 4 phyla: Firmicutes, Bacteroidetes, Actinobacteria and Proteobacteria. However, while the nasal microbiota consists primarily of members of the phylum Actinobacteria and, to a lesser extent, of the phylum Firmicutes^20^, the most abundant DNA sequences in the OE associated microbiota belonged to the phyla Firmicutes and Bacteroidetes. This distinct phylum-level distribution pattern supports a niche-specific colonization. Nevertheless, we cannot rule out that these distribution differences may be due to host species specificities, as our study was performed in mice and not in humans. For instance, it has been reported that Proteobacteria make up the major constituents of the mouse lung microbiota^22^, which is not the case in humans^21^. We also cannot exclude that the actual differences are less marked than reported here, as marker genes amplification-based surveys are subject to a variety of biases. Nevertheless, distribution at the family level within the major phyla was characterized by the marked prevalence of few families. This was striking in the Bacteroidetes phylum, where *Bacteroidaceae* accounted for a large percentage of the DNA sequences in 11 out of 12 mice, and in the Actinobacteria phylum, where *Bifidobacteriaceae* were largely predominant in the same 11 mice. *Enterobacteriaceae* was the most common Proteobacteria family, largely prevalent in 6 out of 12 mice, while the distribution was more balanced in the Firmicutes phylum, with mainly *Enterococcaceae*, *Lachnospiraceae* and *Ruminococcaceae* in various proportions across individuals. A similar limited variation at the family-level was previously observed in human nares, with a dominant position of *Propionibacteriaceae* among Actinobacteria, and of *Lachnospiraceae* and *Staphylococcaceae* among Firmicutes^23^. This is unlike gut microbiota, whose family diversity within the main Firmicutes, Bacteroidetes, Actinobacteria and Proteobacteria phyla, is much greater^24^. As reported in all explored body habitats of humans, including gut, skin, nares, or oral cavity^6^, the relative proportions of phyla and families within the OE microbiota displayed interindividual variations, with some individuals showing a greater diversity and more balanced proportions than others. Factors driving such differences remain to be identified, since all mice were inbred individuals of the same strain, sex and age, housed in the same environmental conditions and fed on the same diet.

In the intestinal epithelium of germfree mice, the villus thickness is reduced because of the lower cellular turn-over^25^. In germfree mice, while the total OE thickness was not altered, the decreased adenylate cyclase III staining indicated a smaller thickness of the cilia layer. Indeed, adenylate cyclase III is mainly expressed in cilia and we observed a similarly reduced layer of cilia using transmission electron microscopy (Fig. 2A). We cannot completely rule out that this reduction is related to the observed lower cellular turn-over. However, this is unlikely as very young olfactory neurons do not possess cilia^26^. Thus, with a decreased cellular turn-over in germfree animals, we should observe a thicker cilia layer due to the higher prevalence of older OSN. The higher amplitude of EOG signals (Fig. 3) coupled with a reduction of olfactory transduction expression genes (Fig. 4A,B) could help to understand this discrepancy. Indeed, this lower expression is consistent with a thinner cilia layer, requiring fewer proteins of the transduction pathway to be synthetized. Furthermore, the thinner cilia layer along with the increase of EOG signal amplitude indicates that the odorant detection per cilium was improved in germfree mice. As the level of cAMP is highly correlated to the length of cilia^27^,^28^, it may be that cilia length is reduced in germfree animals because the olfactory transduction pathway is more efficient. Finally, we can rule out that our observation could be based solely on a reduction of OSN numbers in germfree mice. Even if the OSNs population is diminished in germfree mice, if their properties did not change, the relative level of expression of their genes should not be altered. Thus, a simple diminution in OSN population size could not explain the observed decreased in gene expression level of olfactory transduction pathway actors that are quantified relative to β-tubIII, a marker of OSN population^29^. Furthermore, if there was a decrease in the number of OSNs, we should observe an increased level of apoptosis in the OE of germfree mice, which we did not. In addition, part of our results argues that the OSN population in germfree mice is not diminished: the amplitude of odorant responses recorded by electrophysiology was mostly stronger in germfree mice and not different for a few odorant, indicating that there is specific alteration of odorant responses which is not compatible by a simple change in global OSN population size.

The mucus layer of the intestinal epithelium is strongly affected by the absence of microorganisms, although no clear explanation exists^25^. In the OE, as odorants need to be solubilized in the mucus layer before they can interact with olfactory receptors, we can expect that the absence of microbiota alters the kinetics of odorant arrival. Indeed, in germfree animals, most odorants activate the OSN populations faster as reflected by shorter rise times in EOG recordings (Fig. 3C and supplementary Table 1). Such accelerated odorant detection is consistent with the absence of microbiota, which could present a barrier between odorants and olfactory cilia. Faster odorant detection is also consistent with the observed higher expression of olfactory binding proteins (Fig. 4C) that are primarily thought to aid the transport of odorants from air through the mucus^9^.

The influence of microbiota on xenobiotic metabolism genes expression has already been observed with a tendency for higher expression in germfree animals, especially in the colon^30^,^31^. The OE possesses a strong xenobiotic metabolism in order to clear odorants from the mucus and to allow continuous analysis of incoming odours^9^. We observed a lower level of expression of detoxifying enzyme genes in the germfree mice OE (Fig. 4D). As those enzymes are responsible for odorant degradation, such decrease could potentially lead to prolonged duration of responses to odorant stimulation. On the contrary, the faster repolarizing kinetics observed with most odorants in germfree animals indicate that OSNs stop responding to the odorant stimulation faster. We can speculate that, when bacteria are present in the OE mucus, they produce metabolites detected by OSNs as odorants. Such metabolites will compete with external odorants as substrates of the epithelial detoxifying enzymes. Thus, they must be cleared continuously in order for the odorant detection to take place properly. In the absence of microbial metabolites, less detoxifying enzymes may be required to clear odorants from the olfactory mucus.

We found that the OE cellular turn-over is reduced in germfree conditions as already observed for the intestinal epithelium^32^. Many previous studies have focused on OE cellular turn-over modulation, showing for example that this turn-over is reduced following nasal occlusion^33^. Nasal occlusion studies are often used to characterise activity driven neuronal survival^34^ but the air flow change induced by this closure may impact the local microbiota. We can also expect that the OE associated microbiota will not be homogeneously distributed among the turbinates. Indeed, as also observed for lung microbiota^21^, those located in posterior areas should hold smaller bacterial populations as they are further away from the airway entrance. Interestingly, the cellular turn-over is slower in the posterior part of the OE^35^. However, our microbiota analysis did not allow to specify how the microbiota is distributed in the different OE areas, since the overall OE was necessary to reach the minimal DNA amount required for analysis.

When considering the influence of microbiota in animal physiology, most studies focus on gut microbiota and its influence on the gastro-intestinal system, because of its proportional importance^19^. However, when considering the recent findings on the impact of microbiota on brain and behaviour, it may be worth paying more attention to changes induced in olfactory functions, especially when working with rodents. Indeed, germfree rodents have been shown to behave differently to conventionally reared conspecifics, with altered anxiety-like behaviour in response to stress factors^36^,^37^,^38^,^39^ and a lower level of social motivation^40^,^41^. The extent to which these behavioural changes are due to alterations in the olfactory capacity of germfree rodents has now to be considered as olfaction is of major importance in rodent behaviour.

Finally, a high variability has been found between individuals in human nasal microbiota^5^,^23^. If OE physiology is differentially impacted by differences in nasal microbiota, it would be very interesting to link disturbance of nasal microbiota with alteration in eating behaviour, which is known to be closely linked to olfaction in humans^42^.

## Material and Methods

### Animals

The germfree C3H/HeN mice were born from germfree parents originating from the germfree breeding facility of Anaxem (Germfree animal facilities of INRA, UMR1319 Micalis). The germfree C3H/HeN colony maintained in the germfree breeding facility of Anaxem was derived from a specific pathogen free C3H/HeN colony through Caesarean section of pregnant females^3^. The conventional, specific pathogen free, C3H/HeN mice were born from specific pathogen free parents purchased from Janvier Labs (St Berthevin, France). The parents were housed and mated in non-sterile isolators in Anaxem to ensure that the conventional offspring used in the experiment was born and raised in the same environmental conditions as the germfree offspring, apart from the microbiota. The germfree experimental mice were then housed throughout the experiment in sterile isolators (Getinge, Les Ulis, France) and the germfree status was monitored weekly by microscopic examination and aerobic and anaerobic cultures of samples of freshly voided feces. The conventional experimental mice were also housed in isolators (non-sterile) to ensure the same sensorial environment between conventional and germfree animals. Within isolators, mice were kept in standard cages containing sterile beddings made of wood shavings; they were given free access to autoclaved tap water and a γ-irradiated (45 kGy) standard diet (R03; Scientific Animal Food and Engineering, Augy, France). The animal room was maintained at 20–24 °C and on a 12-h light/dark cycle (lights on at 7:30 am). All mice (mixed gender) were used at 8–10 weeks of age. They were euthanised by decapitation following sodium pentobarbital anaesthesia and heads were removed to perform OE analyses. Procedures were carried out in accordance with the European guidelines for the care and use of laboratory animals; they were approved by the Ethics Committee of AgroParisTech and the INRA Research Center of Jouy-en-Josas (approval reference: 14, 15, 16, 17, 18, 19, 20, 21, 22, 23, 24, 25, 26, 27, 28, 29, 30, 31, 32, 33, 34, 35, 36, 37, 38, 39, 40, 41, 42).

### Metagenomic DNA extraction, 16S rRNA gene sequencing and bioinformatic analysis

For the extraction of the metagenomic DNA, the whole olfactory mucosa was collected and the DNA purified following the method of Godon *et al.*^43^ with slight modifications. The V3-V4 region of the 16S rRNA gene was amplified from purified metagenomic DNA with the universal primers F343 (CTTTCCCTACACGACGCTCTTCCGATCTACGGRAGGCAGCAG) and R784 (GGAGTTCAGACGTGTGCTCTTCCGATCTTACCAGGGTATCTAATCCT), using 30 amplification cycles with an annealing temperature of 65 °C. The amplicon lengths were about 510 bp (the exact length varies depending on the species). Because MiSeq sequencing enables paired 250-bp reads, the ends of each read overlap and can be stitched together to generate extremely high-quality, full-length reads covering the entire V3-V4 region. Single multiplexing was performed using a home-made 6 bp index, which was added to the R784 primer during a second PCR with 12 cycles using the forward primer (AATGATACGGCGACCACCGAGATCTACACTCTTTCCCTACACGAC) and the modified reverse primer (CAAGCAGAAGACGGCATACGAGAT-index-GTGACTGGAGTTCAGACGTGT). The resulting PCR products were purified and loaded onto the Illumina MiSeq cartridge according to the manufacturer instructions. The quality of the run was checked internally using PhiX, and each pair-end sequence was assigned to its sample using the previously integrated index. Bioinformatic analysis started by trimming the sequences for adaptors and assembling them with Flash1.6.2^44^. PCR primers were removed and sequences with sequencing errors in the primers were excluded (Mothur)^45^. We rarefied all samples to the same depth (10 221, the minimal sample depth). Chimera were removed with Uchime^46^ and Mothur. Reads were clustered into Operational Taxonomic Units (OTUs) at the 97% identity level using Esprit-tree^47^. We picked a reference sequence for each OTU and assigned it at different taxonomic levels (from phylum to species) using the Greengenes database (release 13-5)^48^ and the RDP classifier^49^.

### Immunohistochemistry and OE thickness measurement

Immunohistochemistry from OE tissue sections was performed as described previously^50^. Briefly, the nasal septum and turbinates were removed as a block and post-fixed overnight at 4 °C in 4% paraformaldehyde PBS. Blocks were cryoprotected with sucrose (30%) and cryo-sectioned sagitally (14 μm thickness). Sections were kept frozen at −80 °C until use. For PCNA staining, we performed an antigen retrieval in a citrate buffer (pH = 6) at 95 °C for 30 min. Non-specific staining was blocked by incubation with 10% non-immune goat serum diluted in PBS containing 2% bovine serum albumin and 0.3% Triton X-100. The sections were then incubated overnight with primary antibodies directed against adenylate cyclase III (ACIII; 1: 2000; rabbit polyclonal, Santa Cruz Biotechnology); cleaved caspase 3 (1: 400; rabbit polyclonal, Cell Signaling; Ozyme, France) or PCNA (1: 200; mouse monoclonal PC10, GeneTex; Tebu-bio SAS, France). Fluorescence staining was performed with Alexa-Fluor-488-conjugated goat secondary antibodies (1: 1000; Molecular Probes; Invitrogen, France). Except for PCNA, sections were finally stained with 2 μg/mL Hoechst 33342 for 10 min and mounted in Vectashield after extensive wash in PBS.

Immunohistochemistry was performed on four transversal sections per mouse, spread regularly through the OE. For all sections, we took four images located dorso-medially at the base of the septum corresponding to zone 1 as defined previously^10^. Images were taken blindly of the animal origin at x100 magnification using a DMBR Leica microscope equipped with an Olympus DP-50 CCD camera using CellF software (Olympus Soft Imaging Solutions GmbH, OSIS, Münster, Germany), except for cleaved caspase 3 images acquired with an Olympus IX71 inverted microscope equipped with an Orca ER Hamamatsu cooled CCD camera (Hamamatsu Photonics France, Massy, France).

Images were quantified using ImageJ (Rasband, W.S., ImageJ, U. S. National Institutes of Health, Bethesda, Maryland, USA, http://imagej.nih.gov/ij/, 1997–2012) to threshold specific ACIII; cleaved caspase 3 and PCNA staining^50^. The same threshold was applied for all images arising from the same experiment. The area of the OE was measured from the Hoechst staining for ACIII and cleaved caspase 3 signal quantification. As the antigen retrieval treatment required for PCNA staining does not allow a sufficient nuclear staining, we used the auto fluorescence of the OE at 555 nm excitation to measure its area. Those measurements allowed us to quantify the percentage of ACIII, cleaved caspase 3 and PCNA staining area in the OE reflecting respectively the cilia layer thickness, the level of apoptosis, and the level of proliferation in the OE.

For the OE thickness quantification, we used the Hoechst staining images and performed 10 measurements spread along the OE for each image. All results were expressed as a relative value of the mean of the conventional group.

### Transmission electron microscopy (TEM)

Samples were fixed with 2% glutaraldehyde in 0.1 M Na cacodylate buffer pH 7.2 at room temperature for 4 hours, and then processed in a micro-wave machine (KOS – Microm Microtech, France); contrasted with Oolong Tea Extract (OTE) 0.5% in cacodylate buffer and then postfixed with 1% osmium tetroxide containing 1.5% potassium cyanoferrate; gradually dehydrated in ethanol (30% to 100%) followed by 2 baths of acetone and embedded in Epon (Delta microscopie, Labège, France).

Thin sections (70 nm) were collected onto 200 copper-mesh grids, and counter stained with lead citrate before examination with a Hitachi HT7700 (Elexience) electron microscope operated at 80 kV. Microphotographs were acquired with a charge-coupled device camera AMT.

### Electroolfactogram recording (EOG)

To compare the global responses of OSNs from germfree and conventional mice, EOG recordings were made from the OE in an opened nasal cavity configuration as described earlier^51^. Mice were euthanized during the light phase (09:00–18:00) to allow continuous recordings during the working day. We took care to alternate them according to their treatment to limit any circadian bias. The hemi-head was placed in a recording chamber under an upright Olympus SZ51 stereo microscope (Olympus, Rungis, France) equipped with a low magnification objective (0.8 to 4x) and two MX-160 micromanipulators (Siskiyou, Inc., Grants Pass, OR, USA). The odour stimulation device was modified from Scott and Brierley^52^. The hemi-head was kept under a constant flow of humidified filtered air (~1000 ml/min) delivered through a 9 mm glass tube. This tube was positioned 2 cm from the epithelial surface. Odour stimulations were performed by blowing air puffs (200 ms, 200 ml/min) through an exchangeable Pasteur pipette enclosed in the glass tube containing a filter paper impregnated with 20 μL of odorant (Sigma Aldrich, Saint-Quentin Fallavier, France). We used the following dilution in mineral oil of odorants (volume/volume): Heptanal ranging from 1:100000 to 1:10; Limonene ranging from 1:1000 to 1:10; Acetophenone at 1:1000 and 1:10; Heptanoic acid, Isoamyl acetate, Octanol, Pyridine, S-Methyl thiobutanoate, and Thiazole at 1:10. EOG voltage signals were recorded using an XtraCell 2 channels amplifier (DIPSI, Chatillon, FRANCE) used in a DC current-clamp configuration (I = 0), low-pass bessel filtered at 1 kHz and digitized at a rate of 2 kHz using a Digidata 1322a A/D converter (Axon Instruments, Molecular Devices, Union City, CA, USA) interfaced to a Pentium PC and Pclamp 9.2 software (Axon Instruments). A reference Ag/AgCl electrode was placed on the frontal bone overlaying the olfactory bulb. Recordings were made with glass micropipettes of 4–5 MΩ filled with a saline solution. EOG were recorded from the center of turbinates IIb and III. These positions gave robust, reproducible and long-lasting EOG recordings ranging from 12 to 20 mV when stimulated with acetophenone 1:1000. Odorant-free air stimulation (with mineral oil) always produced signals around 1 mV amplitude. Analyses were performed using Clampfit 9.2 (Axon Instruments) to measure peak amplitude, rise time (from 10% to 90%), as well as fast (from 100% to 70%) and slow (from 40% to 10%) decay times of EOG responses. Since the EOG response kinetics correlated highly with the amplitude, the rise time and the two decay slopes were normalized to the corresponding response peak amplitude prior to statistical analysis.

### qPCR

Total RNA was extracted from frozen olfactory mucosa using the Trizol method. OligodT first strand cDNA were synthesized from 5 μg total RNA by the Superscript II reverse transcriptase (Invitrogen) following the manufacturer recommendations, then treated with DNase I. For quantitative PCR, 5 μl of 125-fold diluted cDNA templates were added to the 15 μl-reaction mixture containing 300 nM primers (sequences in Supplementary Table 2)and SYBR Green GoTaq^®^ qPCR Master Mix (Promega, Charbonnières, France). The PCR was performed on a Mastercycler^®^ ep realplex (Eppendorf) during 40 amplification cycles consisting of 45 s at 94 °C, 45 s at 60 °C and 45 s at 72 °C. Quantification was achieved using the ΔΔCt method. mRNA expression was normalized to the expression level of either the OSN specific β-tubulin III for OSN expressed genes^29^ or β-actin for other genes. An efficiency corrective factor was applied for each primer pair.

### Statistical analysis

Data are expressed as mean ± standard error of the mean (SEM). The nonparametric Mann–Whitney two-tailed test was used to determine statistical significance of differences between groups. A probability value of at least *P* < 0.05 was used as an indication of significant differences.

## Additional Information

**How to cite this article**: François, A. *et al.* Olfactory epithelium changes in germfree mice. *Sci. Rep.* **6**, 24687; doi: 10.1038/srep24687 (2016).

## Supplementary Material

Supplementary Information
