# Supplementary material for: Olfactory epithelium changes in germfree mice
Source: Sci Rep. 2016 Apr 19;6:24687. doi: 10.1038/srep24687 (PMC4835764; doi:10.1038/srep24687)
Supplement: Supplementary Information [file srep24687-s1.doc]

# Olfactory epithelium changes in germfree mice

Adrien François^1^, Denise Grebert^2^, Moez Rhimi^3^, Mahendra Mariadassou^4^, Laurent Naudon^5^, Sylvie Rabot^3^, Nicolas Meunier^1*^

^1^NBO, UVSQ, INRA, Université Paris-Saclay, Jouy-en-Josas, F-78350 France; ^2^NBO, INRA, Université Paris-Saclay, F-78350 Jouy-en-Josas, France; ^3^Micalis Institute, INRA, AgroParisTech, Université Paris-Saclay, F-78350 Jouy-en-Josas, France; ^4^INRA, UR1404 MaIAGE, F-78350 Jouy-en-Josas, France; ^5^Micalis Institute, INRA, AgroParisTech, CNRS, Université Paris-Saclay, F-78350 Jouy-en-Josas, France

*Correspondance:

Dr. Nicolas Meunier

NBO, UVSQ, INRA, Université Paris-Saclay, Jouy-en-Josas, F-78350 France

Tel: 33 (0)1 34 65 25 55

Email: [nicolas.meunier@jouy.inra.fr](mailto:nicolas.meunier@jouy.inra.fr)

**Table 1: Differences of EOG signals characteristics between conventional and germfree animals**

| Odorant | | | Dilution in mineral oil | Peak  (mV) | Area  (V.ms) | Rise time  (ms/mV) | Decay time^Fast^ (ms/mV) | Decay time^Slow^ (ms/mV) |
| --- | --- | --- | --- | --- | --- | --- | --- | --- |
|  |  | |  |  |  |  |  |  |
|  | Acetophenone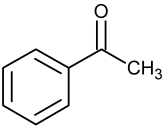 | | 0.001 | 15.73 *vs* 16.5 ns | 61.7 *vs* 79.5 ns | 8.4 *vs* 7.8 ns | 22 *vs* 25.4  * | 691 *vs* 751 ns |
|  |  |  | 0.1 | 11.93 *vs* 13.08 ns | 143.3 *vs* 122.9 ns | 11.5 *vs* 11.5 ns | 43.0 *vs* 44.9 ns | 3085 vs 1828 * |
|  | Heptanal  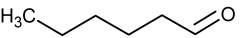 | | 0.00001 | 2.76 *vs* 5.13 *** | 1.4 *vs* 2.6  ns | 79.6 *vs* 29.5  * | 42.7 *vs* 20.7  ** | 190 *vs* 93  * |
|  |  |  | 0.0001 | 8.93 *vs* 11.33  ****** | 6.7 *vs* 8.7  * | 15.4 *vs* 10.5  * | 17.4 *vs* 14.3  ** | 94 *vs* 74  ns |
|  |  | | 0.001 | 15.45 *vs* 18.63  ****** | 18.9 *vs* 25.8  ** | 7.2 *vs* 5.8  * | 15.1 *vs* 13.1  * | 98 *vs* 99  ns |
|  |  | | 0.01 | 19.53 *vs* 22.59 ******* | 44.3 *vs* 49.9 ns | 4.8 *vs* 3.9  * | 20.7 *vs* 19.1  ns | 172 *vs* 139  ns |
|  |  | | 0.1 | 23.11 *vs* 26.16 ns | 145.0 *vs* 164.4 ns | 4.8 *vs* 3.5  *** | 36.8 *vs* 35.2  ns | 539 *vs* 386  ns |
|  | Heptanoic acid  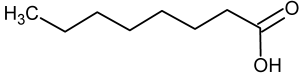 | | 0.1 | 8.63 *vs* 11.82 ******* | 5.3 *vs* 7.8  ** | 17.0 *vs* 11.2  ** | 16.9 *vs* 13.5  ** | 69 *vs* 50  ns |
|  | Isoamyl acetate  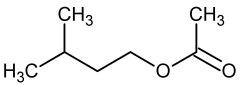 | | 0.1 | 17.45 *vs* 20.28 ****** | 65.8 *vs* 79.7  * | 6.5 *vs* 5.2  ** | 27.1 *vs* 26.2  ns | 409 *vs* 336  * |
|  | Limonene  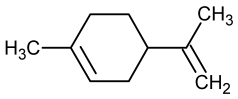 | | 0.01 | 5.93 *vs* 7.45  * | 4.9 *vs* 5.7  ns | 27.9 *vs* 22.2  * | 28.0 *vs* 19.9  ** | 207 *vs* 142  ** |
|  |  |  | 0.1 | 11.7 *vs* 14.37  * | 19.5 *vs* 19.9  ns | 15.1 *vs* 11.2  * | 22.7 *vs* 14.9  * | 283 *vs* 177  * |
|  | Octanol  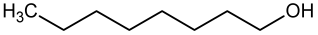 | | 0.1 | 15.6 *vs* 17.9  ** | 103.4 *vs* 117.7 ns | 8.6 *vs* 6.8  ** | 25.8 *vs* 24.4  ns | 1180 *vs* 1094  ns |
|  | Pyridine | 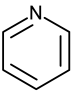 | 0.1 | 12.96 *vs* 13.1  ns | 138.1 *vs* 95.6  ns | 14.1 *vs* 11.9  ns | 55.8 *vs* 42.9  ns | 1876 *vs* 1391  * |
|  | S-Methyl thiobutanoate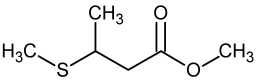 | | 0.1 | 20.4 *vs* 22.99  * | 60.2 *vs* 68.6  ns | 5.0 *vs* 4.0  ** | 25.8 *vs* 22.9  ns | 221 *vs* 177  ns |
|  | Thiazole | 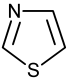 | 0.1 | 12.6 *vs* 15.57 ****** | 71.4 *vs* 139.9 ******* | 14.6 *vs* 13.4  ns | 38.4 *vs* 41.7  ns | 1215 *vs* 1517  ns |
|  |  | |  |  |  |  |  |  |

**P*<0.05; ***P*<0.01; ****P*<0.001

**Table 2: Primers used to perform qPCR analyses**

| **Gene** | **Sense primer** | **Antisense primer** | **Amplicon size (bp)** |
| --- | --- | --- | --- |
| *Accession number* |
| **PCNA** | 5' - TCACAAAAGCCACTCCACTGTCTC | 5' - CTGGCATCTCAGGAGCAATCTTCA | 198 |
| *NM_011045.2* |
| **KI67** | 5' - CACAACCAGAAAGTGCCATAACCC | 5' - GGCATACCAGCTGTTGAATCCAGT | 211 |
| *NM_001081117.2* |
| **Olfr2** | 5' - CATTGCTCAATCCCATCATCT | 5' - TGCTGGATTTCTTGGTATTGG | 111 |
| *NM_010983.2* |
| **Olfr937** | 5' - TCTGCCATTCCACTGTCATTACCC | 5' - AGGGGTTACAGATGGCAACATAGC | 177 |
| *AY318125.1* |
| **Olfr151** | 5' - GTAGGACTCATCGGTTCAGCA | 5' - GGGGAGGATGTCACAGAAGTA | 96 |
| *NM_207664.2* |
| **Golf** | 5' - GCATCTGGAATAACAGGTGGTT | 5' - GGCATTACTCCGGGAAATAGTCT | 125 |
| *AY179169.1* |
| **ACIII** | 5' - TTGACTCTCTCCTGGACAATCC | 5' - CTTGTAAAGCCATTGGTGTTGA | 118 |
| *NM_138305.3* |
| **Cnga2** | 5' - GTGGAACTGGTACTGAAGCTTCGT | 5' - TGACAGCAAGGCATACTGAGTCAC | 153 |
| *NM_007724.2* |
| **PDE1C2** | 5' - CTGGAGGGAGTTTCGAACTTTGGT | 5' - GCTTTCGCAGGATGACTGATGTCT | 174 |
| *NM_001025568.2* |
| **CYP1A2** | 5' - TCCAGGAGCACTACCAAGACTTCA | 5' - ACGTTAGGCCATGTCACAAGTAGC | 211 |
| *NM_009993.3* |
| **CYP2A5** | 5' - GCCCAAGTATGAGGACCGAATGAA | 5' - CAGAGCCCAGCATAGGAAACACTT | 173 |
| *NM_007812.4* |
| **CYP2G1** | 5' - TACACTGACGCTGTCATCCATGAG | 5' - TCTGGGTAGCGGAAGTATTTGGGA | 176 |
| *L81171.1* |
| **GSTA3** | 5' - ACTGGTGCAGACCAAAGCCATT | 5' - CAGGGAAGTAACGGTTCCTGGTTT | 212 |
| *NM_001077353.2* |
| **UGT2A1** | 5' - ACACGATGACAAGCTCAGATCTGC | 5' - TTGGCTCCTTTGTGACGCATGA | 169 |
| *NM_053184.2* |
| **OBP1a** | 5' - GACATTTGGACTGGCACATGCT | 5' - CCAGTGATTGTGGTCAATGAGC | 201 |
| *NM_008754.2* |
| **OBP1b** | 5' - TGGTATTTGGACTGGCCCATGT | 5' - CCAGTGACTGTGGTCAATGAGCA | 220 |
| *XM_006544587.1* |
| **β-tub III** | 5' - TGAGGCCTCCTCTCACAAGT | 5' - GGCCTGAATAGGTGTCCAAA | 105 |
| *NM_139254.2* |
